# Diagnostic accuracy of nanopore sequencing for the rapid diagnosis of pulmonary tuberculosis: A protocol for a systematic review and meta-analysis

**DOI:** 10.1371/journal.pone.0304162

**Published:** 2024-06-06

**Authors:** Guocan Yu, Xudong Xu, Yanqin Shen, Bifei Fang

**Affiliations:** 1 Department of Thoracic Surgery, Hangzhou Red Cross Hospital, Hangzhou, Zhejiang, China; 2 Department of Nursing, Hangzhou Red Cross Hospital, Hangzhou, Zhejiang, China; Gulu University, UGANDA

## Abstract

**Background:**

Pulmonary tuberculosis (PTB) is the most common type of tuberculosis (TB). Rapid diagnosis of PTB can help in TB control. Although the use of molecular tests (such as the GeneXpert MTB/RIF) has improved the ability to rapidly diagnose PTB, there is still room for improvement. Nanopore sequencing is a novel means of rapid TB detection. The purpose of this study was to establish a systematic review and meta-analysis protocol for evaluating the accuracy of nanopore sequencing for the rapid diagnosis of PTB.

**Methods:**

We completed this protocol according to the Preferred reporting items for systematic review and meta-analysis protocols (PRISMA-P) statement and registered on the PROSPERO platform. We will screen studies related to nanopore sequencing for diagnosis of PTB by searching through PubMed, EMBASE, the Cochrane Library using English, and Wanfang database, CNKI (China National Knowledge Infrastructure) using Chinese. Eligible studies will be screened according to the inclusion and exclusion criteria established in the study protocol. We will evaluate the methodological quality of the individual included studies using Quality Assessment of Diagnostic Accuracy Studies (QUADAS-2). We will use Stata (version 15.0) with the *midas* command and RevMan (version 5.3) for meta-analysis and forest plots and SROC curves generation. A p < 0.05 was treated as a statistically significant difference. When significant heterogeneity exists between studies, we will explore sources of heterogeneity through meta-regression analysis and subgroup analysis.

**Conclusion:**

To the best of our knowledge, this will be the first systematic review and meta-analysis of nanopore sequencing for the diagnosis of PTB. We hope that this study will find a new and effective tool for the early diagnosis of PTB.

**PROSPERO Registration number:**

CRD42023495593.

## Introduction

Tuberculosis (TB), a chronic infectious disease caused by *Mycobacterium tuberculosis* complex (MTB) infection of the body, continues to pose a major threat to human health [1]. TB causes millions of human deaths each year, the most deaths caused by a single infectious disease [2]. Pulmonary TB (PTB) is the most predominant type of TB, accounting for about 80% of all TB [3]. Because of the open nature of the respiratory tract, MTB can be discharged to the outside world via the respiratory tract and thus infect other people; therefore, PTB is the main source of infection for TB transmission [4]. The control of PTB is the key to ending TB epidemic [5]. The control of PTB relies on early and rapid diagnosis of the disease so that standardized and effective treatment can be given to minimize the number and duration of MTB emissions in patients with PTB [3].

The traditional classical TB detection means have some limitations. The acid-fast bacilli (AFB) smear is a very widely used routine test for TB, although it is simple to operate and inexpensive, it has low sensitivity, cannot effectively detect PTB [3]. AFB smear cannot distinguish between MTB and non-tuberculosis mycobacteria (NTM) [6], and there is a possibility of miss-diagnosis and misdiagnosis [6]. MTB culture is another test that is essential for the diagnosis of PTB, and while it is effective in diagnosing PTB and differentiating NTM, the time it takes to obtain results is lengthy, often taking weeks [7], and this time may also lead to significant MTB dissemination [8]. GeneXpert MTB/RIF is a commonly used molecular diagnostic tool in clinical practice [9], but the test remains limited in its ability to diagnose smear-negative PTB [2], and it only detects rifampicin resistance [10]. The development of other effective early rapid tests for PTB diagnosis is urgently needed.

Gene sequencing is the gold standard for the diagnosis of infection pathogens, by which the species of the pathogen can be clarified [11]. GeneXpert MTB/RIF has played a very important role in the rapid diagnosis of PTB, but its diagnostic accuracy still needs to be further improved. Several studies have shown that GeneXpert MTB/RIF is less accurate than gene sequencing methods (such as nanopore sequencing) for the diagnosis of PTB [12–14]. Nanopore sequencing is a new generation of gene sequencing technology, which is characterized by fast sequencing speed, long sequencing reads, real-time monitoring of sequencing data, and portable equipment [15]. So far, nanopore sequencing is relatively expensive compared to other tests and requires good laboratory conditions, but these are not worth mentioning compared to its benefits [16]. Nanopore sequencing is now showing its advantages in the field of infectious disease diagnosis, which can effectively improve the detection efficiency of pathogenic bacteria [17, 18]. Nanopore sequencing has also been applied in the early and rapid diagnosis of TB, showing satisfactory diagnostic accuracy and is a new star in the field of TB diagnosis [12]. In PTB, even in smear-negative PTB diagnosis, nanopore sequencing likewise demonstrated very good diagnostic accuracy [13, 19]. The results of previous research suggested that the area under the curve (AUC) value obtained via nanopore sequencing were significantly higher than the AUC value obtained via AFB smear microscopy (0.88 vs. 0.55; P < 0.001), MTB culture (0.88 vs. 0.68; P < 0.001), and GeneXpert MTB/RIF (0.88 vs. 0.71; P < 0.001) [20]. Studies on nanopore sequencing for PTB are basically single-center studies [13, 21], and although the performance is satisfactory, there is no evidence-based medical evidence to evaluate the overall diagnostic accuracy of nanopore sequencing for PTB. The objective of this study is to determine the accuracy of nanopore sequencing on the diagnosis of PTB and whether that effect could improve the diagnosis of PTB as a whole using a systematic review and meta-analysis approach, enabling clinicians to better recognize the role of nanopore sequencing in the rapid diagnosis of PTB.

## Methods

### Design and registration

We completed this protocol for systematic review and meta-analysis according to the Preferred reporting items for systematic review and meta-analysis protocols (PRISMA-P) statement [22] and registered on the PROSPERO platform with registration number CRD42023495593. We will report the full study results according to the Preferred Reporting Items for Systematic Reviews and Meta-Analysis for Diagnostic Test Accuracy (PRISMA-DTA) statement [23]. Ethical approval is not required for systematic reviews and meta-analyses; therefore, this study was exempt from ethical approval.

### Information sources

We will screen studies related to nanopore sequencing for diagnosis of PTB by searching through PubMed, EMBASE, the Cochrane Library using English, and Wanfang database, CNKI (China National Knowledge Infrastructure) using Chinese. References to published meta-analysis or reviews on similar topics will be carefully evaluated to further identify suitably qualified studies.

### Search strategy

Two independent researchers (Guocan Yu and Xudong Xu) will design a search strategy for each database that meets the needs of the topic and finalize the search strategy after discussing with each other. The two researchers will develop an effective search strategy through consultation. We will search English databases in English and Chinese databases in Chinese, and there will be no time restriction on the search process. We will conduct an update search prior to the completion of the research to minimize the omssion of studies that might meet the inclusion criteria. The search strategy for PubMed is reported as follows:

#1 "Tuberculosis, Pulmonary"[Mesh] OR “Tuberculoses, Pulmonary” OR “Pulmonary Tuberculoses” OR “Pulmonary Tuberculosis” OR “Pulmonary Consumption” OR “Consumption, Pulmonary” OR “Consumptions, Pulmonary” OR “Pulmonary Consumptions” OR “Pulmonary Phthisis” OR “Phthises, Pulmonary” OR “Phthisis, Pulmonary” OR “Pulmonary Phthises” OR PTB OR “Pulmonary TB” OR PTB
#2 "Nanopore Sequencing"[Mesh] OR “Nanopore Sequencings” OR “Sequencing, Nanopore” OR “third generation sequencing” OR “Oxford Nanopore Technology” OR “Oxford Nanopore Technologies” OR ONT
#3 #1 AND #2

### Eligibility criteria

#### Type of studies

Any study that reported results on the diagnostic accuracy of nanopore sequencing for the diagnosis of PTB will be included in this study.

#### Participants

Patients with PTB, there will be no restrictions on age, race, or gender.

#### Index tests

Nanopore sequencing will be treated as index test.

#### Comparator test

Comparator tests will be optional as this study only evaluated the diagnostic accuracy of nanopore sequencing for PTB.

#### Outcomes

Diagnostic accuracy metrics such as sensitivity, specificity, positive predictive value (PPV), negative predictive value (NPV) and AUC will be treated as outcome metrics.

#### Reference standard

Clinical diagnosis and MTB culture will be treated as reference standards. Clinical diagnosis is the result of a comprehensive assessment of multiple evaluation indicators (such as clinical symptoms, pulmonary imaging changes, TB immunologic testing, MTB smear, culture, molecular diagnostic test results of respiratory specimens and anti-TB efficacy).

#### Target conditions

Original studies evaluating nanopore sequencing for the diagnosis of PTB and reporting clear reference standards will be included in this systematic review and meta-analysis. Articles for which true positive (TP), false positive (FP), false negative (FN), and true negative (TN) values can be obtained from the original study or calculated from information provided in the article will meet the inclusion criteria. If insufficient information was provided in the study, we will contact the corresponding author of the article to seek additional information. Studies for which TP, FP, FN, and TN values are not available, case reports, reviews, conference abstracts without full-text reporting, and studies published in languages other than English and Chinese will be excluded.

#### Literature screening and selection

We will use Endnote (version 9.2) to unify the management of literature obtained from searches of various databases. We will strictly follow the inclusion and exclusion criteria set by this protocol to screen the literature. Two independent researchers (Guocan Yu and Xudong Xu) will individually screen relevant literature, first by title, then abstract, and finally full text to determine if it fits the research topic. They will check the screening results with each other, and articles with inconsistent conclusions will be further analyzed and discussed with a third independent researcher (Bifei Fang) to make a final decision.

#### Data extraction

The final included studies obtained through screening will be extracted with relevant data such as basic information about the study, nanopore sequencing for diagnosis of PTB. Basic information includes the year the study was published, the country in which the study was conducted, the name of the first author, the type of study, the method of patient selection, the type of patient (adult or children, AIDS or not), the sample size, the type of PTB (smear-positive or not), and reference standards (clinical diagnosis or MTB culture). Information related to nanopore sequencing for diagnosis of PTB, such as TP, FP, FN, TN values, type of samples (sputum or bronchoalveolar lavage fluid), and type of nanopore sequencing (targeted amplification or not). As in the literature screening stage, the same two independent researchers will each extract relevant information and check it with each other, and where there is disagreement discuss it with the third independent researcher for a final decision.

#### Methodological quality assessment

We will evaluate the methodological quality of the individual included studies using Quality Assessment of Diagnostic Accuracy Studies (QUADAS-2) [24]. This is a widely used evaluation tool that incorporates 4 domains (patient selection, index test, reference standard, and flow and timing). The same two independent researchers will assess the methodological quality of the individual included studies separately, and inconsistencies will be decided through discussion with a third independent researcher. We will use funnel plots to examine data to identify the potential for studies to be missing.

#### Data synthesis and statistical analysis

We will calculate the pooled sensitivity, specificity, PPV, NPV, AUC and their 95% confidence intervals (CIs) using the four values TP, FP, FN, and TN. We will calculate and evaluate the relevant outcome metrics separately according to different reference standards. For the evaluation of heterogeneity between studies we will use the I statistic, where I^2^ = 0 suggests that there is no heterogeneity between studies, I^2^<50% suggests that there is insignificant heterogeneity between studies, and I^2^>50% suggests that there is significant heterogeneity between studies. When significant heterogeneity existed between studies, we will explore the source of heterogeneity, which will be further explored through subgroup analysis and meta-regression analysis if the number of included studies are sufficient. We will perform subgroup analysis and meta-regression analysis based on different study types, different methods of patient screening, different types of specimens, different types of patients, different types of PTBs, and different types of nanopore sequencing. The robustness of the correlation analysis will be evaluated through sensitivity analysis. We will use Stata (version 15.0, Stata Corp., College Station, TX, USA) with the *midas* command and RevMan (version 5.3, Cochrane Collaboration, Oxford, United Kingdom) for meta-analysis and forest plots and SROC curves generation [25]. A p-value of less than 0.05 was treated as a statistically significant difference.

#### Evidence evaluation

We will evaluate the quality of evidence by following the Grading of Recommendations Assessment, Development and Evaluation (GRADE)-guidelines [26]. Based on the results of the assessment, the quality of evidence will be categorized into four levels: high, moderate, low, and very low.

## Discussion

With a large patient population, PTB is a major stumbling block on the road to ending TB and requires focused attention [27]. Early and rapid diagnosis is a prerequisite for PTB eradication, and nanopore sequencing may be an effective tool to significantly improve early diagnosis. To the best of our knowledge, this will be the first systematic review and meta-analysis of nanopore sequencing for the diagnosis of PTB. Our study will include only relevant studies in English and Chinese, which may lead to biased results. We will seek to publish our meta-analysis in peer-reviewed journals, and will upgrade the manuscript if there are corrections that need to be made. We hope that this study will find a new and effective tool for the early diagnosis of PTB.

## Supporting information

S1 File Preferred Reporting Items for Systematic review and Meta-Analysis Protocols (PRISMA-P) checklist. (DOC)

S2 File Search strategies for English and Chinese databases. (DOCX)
