# Supplementary material for: Diagnostic accuracy of nanopore sequencing for the rapid diagnosis of pulmonary tuberculosis: A protocol for a systematic review and meta-analysis
Source: PLoS One. 2024 Jun 6;19(6):e0304162. doi: 10.1371/journal.pone.0304162 (PMC11156269; doi:10.1371/journal.pone.0304162)
Supplement: S2 File — (DOCX) [file pone.0304162.s002.docx]

Search

English database

#1 "Tuberculosis, Pulmonary"[Mesh] OR “Tuberculoses, Pulmonary” OR “Pulmonary Tuberculoses” OR “Pulmonary Tuberculosis” OR “Pulmonary Consumption” OR “Consumption, Pulmonary” OR “Consumptions, Pulmonary” OR “Pulmonary Consumptions” OR “Pulmonary Phthisis” OR “Phthises, Pulmonary” OR “Phthisis, Pulmonary” OR “Pulmonary Phthises” OR PTB OR “Pulmonary TB” OR PTB

#2 "Nanopore Sequencing"[Mesh] OR “Nanopore Sequencings” OR “Sequencing, Nanopore” OR “third generation sequencing” OR “Oxford Nanopore Technology” OR “Oxford Nanopore Technologies” OR ONT

#3 #1 AND #2

Chinese database

#1 肺结核 OR 肺痨 OR 肺部结核病 OR 支气管结核 OR 气管内膜结核

#2 纳米孔测序 OR 第三代测序 OR 牛津纳米孔技术

#3 #1 AND #2
